# COVID-19 Vaccination and Mental Disorders, What Has Been Accomplished and Future Direction

**DOI:** 10.3390/brainsci12020292

**Published:** 2022-02-20

**Authors:** Gianluca Pandolfo, Giovanni Genovese, Fiammetta Iannuzzo, Antonio Bruno, Giovanni Pioggia, Sebastiano Gangemi

**Affiliations:** 1 Department of Biomedical and Dental Sciences, Morphological and Functional Images, University of Messina, 98121 Messina, Italy; gpandolfo@unime.it (G.P.); f.iannuzzo@libero.it (F.I.); antonio.bruno@unime.it (A.B.); 2 Institute for Biomedical Research and Innovation, National Research Council of Italy (IRIB-CNR), 98125 Messina, Italy; giovanni.pioggia@cnr.it; 3 School and Operative Unit of Allergy and Clinical Immunology, Department of Clinical and Experimental Medicine, University of Messina, 98125 Messina, Italy; gangemis@unime.it

**Keywords:** COVID-19 vaccination, mental disorders, psychiatry, vaccine hesitancy

## Abstract

The consequences of the pandemic on mental health are among the most important side effects of COVID-19. Wide concerns have emerged both regarding vaccine hesitation in the general population, and the vaccine’s implementation plan. The aim of this study is to evaluate how the scientific community has investigated the relationship between the COVID-19 vaccine and mental disorders. Contrary to expectations, having a full-blown psychiatric pathology seems to positively affect the attitude towards the vaccine, except for PTSD. The intense fear that accompanied the current world emergency has made this pandemic unique; we discuss how it might be one of the factors involved in this result. Further experimental investigations are needed to estimate how personality traits, hyperarousal, and negative emotions influence vaccine compliance both in the general population and in people living with mental disorders.

## 1. Introduction

The prompt development of an anti-coronavirus disease 2019 (COVID-19) vaccine has aroused opposite reactions in the general population with harsh debate between those who favored its use and those who did not, even before its development was completed. The use of social media might have contributed to misinformation [1] rather than emphasizing how this achievement is the culmination of a research path whose foundations can be traced back to 1990 [2], combined with the contemporary effort of dozens of laboratories in the world at a time of crisis. The first two COVID-19 vaccines put at the disposal of health care systems use lipid nanoparticles to deliver antigen mRNA into human cells to induce protein expression. The complexity of the mechanism of action compared to traditional vaccines, the latter closest to an understandable and ancient principle such as similia similibus curantur, led non-experts to look with suspicion at these new technologies, or even at conspiracy theories regarding mass genome editing, highlighting how the pandemic reveals some fundamental conflicts of our time, for instance between ethics and technique.

In this context, concerns regarding both vaccine hesitation in the general population and vaccine implementation have emerged. One of the categories considered to be vulnerable are people with serious mental disorders (SMI), which prevalence is estimated to be between 0.4% and 7.7% [3]. The consequences of the pandemic on mental health are among the most important side effects of COVID-19 [4]. According to a survey by the World Health Organization evaluating the impact of COVID-19 on mental, neurological, and substance use services, the majority of mental health services reported disruptions of essential interventions. In order to respond to decreased volumes both in inpatient and outpatient settings, high income countries have replaced in-person consultations by using telemedicine or helplines [5]. Despite the effort, current research highlights that people with mental disorders are at increased risk of COVID-19 infection and mortality [6,7,8], and the worsening of a diagnosed psychiatric illness [9,10,11], including during adolescence [12]. Furthermore, the expression of psychiatric symptoms in the general population has increased during the COVID-19 pandemic [13,14,15], which might correspond with pandemic trends [13]. In fact, as well as a trigger factor due to the psychological burden of the emergency, the pandemic could represent an opportunity for the emergence of subthreshold mental disorders that were silent within structured daily activities [16].

## 2. Aims

The relationship between COVID-19 vaccination and mental disorders is an important emergent field of study. Although several articles have focused on the neuro-psychiatric implications of the pandemic [17,18], few have investigated the attitudes of people with mental illness towards vaccination. Mazereel et al. [19] did not find articles about this issue at the time of their review and state that research on this topic is urgently needed. Given the novelty of this research topic, the aim of the current study is to report an overview of the articles examining COVID-19 vaccination in relation to mental disorders, and discuss how scientific research has addressed this topic, in order to provide suggestions for further research and direction. We also believe that the current study can help identify useful insights for the management of future pandemic emergencies.

## 3. Attitudes toward Vaccination and Mental Disorders, Studies before COVID-19

Numerous studies have investigated vaccine acceptance or hesitancy in the general population as complex phenomena [20]. Vaccine safety was identified as the largest area of concern [20]. Vaccine acceptance entails trust in the vaccine, the provider, and in the policy-maker, which includes the health system and government [21]. On the other hand, vaccine hesitancy, defined as the delay in acceptance of vaccination despite availability, is associated to confidence, complacency, convenience [22], and socioeconomic determinants [23].

A few studies have investigated psychiatric factors: State anxiety was found to be higher in healthcare workers who evaluate influenza A/H1N1 vaccination as unsafe [24]; Chan et al. found a positive correlation between the anxiety level and the vaccine uptake willingness in Hong Kong’s general population [25]. Moreover, anxious–depressive symptoms were associated with vaccine uptake in pregnant women [26].

According to our knowledge, the first study to explore vaccination among patients diagnosed with mental illness was the study from Lorenz et al. [27]: they observed from 2010 to 2011 a lower rate (28.4%) of vaccination against flu in psychiatric patients compared to the general population. Significant factors associated with the vaccination status were perceived effectiveness, recommendations from healthcare providers, and perception that they can acquire the flu from the vaccine. These results were similar to an interesting systematic review from Schmid et. al. [28] which evaluated evidence about influenza vaccine hesitancy from 2005 to 2016. They proposed a model that takes into account physical, context, and sociodemographic determinants which interact with psychological determinants. Among the psychological factors, the most important appeared to be utility, risk perception, and social benefit, while considerable contextual barriers were access to vaccination, interaction with healthcare system, and reception of a direct recommendation from medical personnel or relatives. Moreover, barriers for seasonal and pandemic influenza uptake were very similar in their study. Maguire et al. [29] investigated the will to adopt protective behaviors in 71 patients with schizophrenia compared to the general population during the influenza pandemic in 2009. Patients with schizophrenia were generally willing to receive vaccination (74%) but significantly less compared with control group (80.1%). Wearing a face mask was the least precautionary measure likely to be adhered to for both groups and it was perceived as minimally effective. In the schizophrenia group, self-efficacy and perceived risk from swine flu were positive predictors of willingness both to wear a face mask and to receive vaccination. The same research group [30] showed that fear was a robust predictor for willingness to adopt all protective measures except for isolation in schizophrenia patients and a weak predictor in the control group. On the other hand, anxiety was associated with a reduced perceived risk in SCZ groups. A recent study from Lawrence et al. based on an elderly sample found that having a mental health diagnosis was associated with greater odds of flu vaccine receipt [31].

## 4. Expert Opinion during COVID-19 Pandemic

We can identify two main phases in research on COVID-19 vaccination and mental disorders: the first phase includes studies before vaccine distribution when the expected lack of vaccines doses made researchers focus mainly on whether to prioritize vaccination for patients with severe mental illness and identify operational guidelines to facilitate the access to care; the second phase includes experimental studies on vaccination and psychopathological variables.

During the first phase the consensus view of researchers was that people with serious mental illness [32,33,34,35,36,37,38] or specific psychiatric disorders (substance use disorders [39,40,41,42], psychosis [43]) needed vaccination priority as others with severe diseases did. The same opinion was stated by the most important psychiatric association, the American Psychiatric Association (APA), which released a COVID-19 guidance manuscript “the role of the psychiatrist in the equitable distribution of the COVID-19 vaccine” [44]. The majority of these research papers were expert opinions based on evidence both from previous data and on data from the current COVID-19 pandemic.

Two manuscripts stated a more controversial position about COVID-19 vaccination in severe psychiatric patients [45,46]. The manuscript from Rehman et al. (2021) [45] was conceptualized at the early stage of pandemic emergency, and highlighted the psychiatric side-effects of medication and possible onset of anxiety, depression, fear, frustration, delusions, hallucination, and anorexia nervosa, based on data on exposure to previous vaccines, while highlighting the importance of psychological intervention. Yang et al. [46] declared the need to be cautious regarding vaccine implementation in high-risk subpopulations such as patients with SMI, given the controversial long term efficacy and safety. This point was well discussed by Toubasi et al. [47], who conducted a meta-analysis based on data from 634,338 COVID-19 patients (10.7% of them were diagnosed with mental disorders). This study showed an increased risk of COVID-19 severity and mortality in this sample; these results leading the authors to recommend vaccination.

Therefore, consistent with the scientific data available at that time, the widespread concern was to not be able to reach this type of patients, with consequences both for people with psychiatric conditions and for the general population. Palermo et al. (2020) [48] in their perspective article highlighted the need for a comprehensive evaluation of physical and psychological profile to improve adherence in frailty patients. One paper [49] focused on the serious difficulties related to the vaccination of people with SMI in a specific geographic area (Latin America). Researchers also developed a plan of action to facilitate vaccination in people with mental illness [39,50]. Interestingly, both studies highlighted the key role of vaccine recommendation from healthcare providers, together with peer support and educational programs, and they suggested providing vaccination within mental health services. Moreover, Salles et al. [51] hypothesized that comorbid insomnia and sleep apnea is a risk factor for reduced response to the COVID-19 vaccination.

## 5. Original Research

During the COVID-19 pandemic, vaccine administration experimental articles that evaluated vaccine adherence/hesitancy in people with mental disorders emerged. The results are reported for major topics and summarized in chronological order in Table 1.

Four articles used assessment instruments: two research groups implemented structured surveys [52,53], the other two used standardized tests to investigate negative affect [55], vaccine hesitancy, and post-traumatic stress disorder (PTSD) symptoms [54]. Caution must be taken in interpreting these results based on a small sample [54,55] or survey-based design [30,37]. Having a full-blown psychiatric pathology seems to positively affect the attitude towards the vaccine [53,55], and vaccine hesitancy does not appear to be a major barrier in this group [52], except for PTSD, associated with higher vaccine hesitancy and higher side effects [54]. “Wearing a mask all the time”, age, and clinically significant anxious depressive symptoms was associated with higher trust in vaccine or willing to pay for it [53,55].

## 6. Conclusions and Future Directions

From a speculative perspective, it is possible that the high values of declared adherence to the COVID-19 vaccine from people with mental disorders, compared with previous vaccination, may be secondary to a greater perception of risks and fear, thanks also to the high levels of general alarm that this pandemic has raised, differently from the previous ones. While functional fear might be related to increased compliance, especially in vulnerable groups [56], fear of the unknown has the potential to convert caution into paranoia and idiosyncratic beliefs in a large percentage of people [57,58]. The management of fear and anxiety should be a core focus of further investigations, implementing self-efficacy and a balanced view of risks. Further research should consider the difference between SMI and state symptomatology, especially when it concerns anxiety–depressive symptoms. Negative affect seems too generic a parameter for an accurate evaluation of the problem, different affect should be evaluated singularly. From another perspective, people with mental disorders might have insight of an implemented vulnerability towards any stressor compared with the general population. The ability of seeking support, which influences adherence during the emergence of acute symptoms, might also be a crucial personality trait for vaccine compliance.

Current evidence highlights that not all psychiatric patients are similar. The hyperarousal associated with PTSD might affect negatively the adherence of this groups of patients; specific interventions that take this into account should be envisaged to increase the efficacy of the vaccination campaign in this subgroup of patients. This study suggests that concerns about people with psychiatric conditions were based, in an early part of the pandemic, mainly on common sense and data on previous pandemics. The intense fear that accompanied the current world emergency has made this pandemic different from the previous ones, with changes of attitudes in the group of psychiatric patients. While psychological variables appear to be similar between general and psychiatric populations, they interact with specific psychopathological dimensions in the latter group. A deeper evaluation of vaccine hesitancy in patients with mental disorders should consider the differences between the distinct diagnostic categories, in order to be able to implement specific intervention measures tailored to the patient’s pathology. It is also important to state that psychological characteristics of people who do not want to get the vaccine cannot be reduced to a psychiatric pathology but are probably more complex and require specific studies with standardized instruments. A comprehensive assessment of the personality, state and trait anxiety, and hyperarousal could be useful for better understanding differences between the general population and psychiatric patients.

## Figures and Tables

**Table 1:** Selected manuscripts evaluating psychiatric factors and COVID-19 vaccination.

Reference	Study Design	Assessment	Main Findings
[52]	Cohort study	Structured online surveys	Vaccine hesitancy does not appear to be a major barrier for vaccine uptake amongst patients with mental illness in Denmark.
[53]	Cross sectional	Structured survey	39.5% trusted that COVID-19 vaccine would be safe and effective. Factors independently associated with trust included age (AOR = 1.03, 95 % CI = 1.02, 1.05, *p* = 0.0001) and wearing a mask all the time (AOR = 2.48, 95 % CI = 1.86, 3.31, *p* = 0.0001).
[54]		ITQ; GAD-7; PHQ-9; vaccine hesitancy (8 items); 23 items indexing severity of COVID-19 vaccine side effect	Participants with clinical PTSD levels showed more anxiety and depressive symptoms, were vaccinated a few days later, showed higher vaccine hesitancy levels, and displayed more severe side effects.
[55]	Cross sectional study	21-item depression, anxiety and stress scale	A significantly higher proportion of people with depression or anxiety disorder (64.5%) were more willing to pay for the COVID-19 vaccine than healthy controls (38.1%) (*p* ≤ 0.001)

Note: SMI = serious mental illness; ITQ = the international trauma questionnaire; GAD-7 = general anxiety disorder-7; PHQ-9 = patient health questionnaire-9; PTSD = post-traumatic stress disorder.

## Data Availability

https://pubmed.ncbi.nlm.nih.gov/ (accessed on 20 September 2021).
